# Immune regulation and clinical response of Chinese herbal injections combined with TACE in hepatocellular carcinoma: a cumulative logit regression and Bayesian network meta-analysis

**DOI:** 10.3389/fmed.2025.1567137

**Published:** 2025-04-04

**Authors:** Wenjuan Gu, Tianyu Zhu, Yujuan Zhang, Ming Xue, Qingfeng Liu

**Affiliations:** ^1^Department of Oncology, Tianjin Kanghui Hospital, Tianjin, China; ^2^Department of Traditional Chinese Medicine, Medical School, Hubei University for Nationalities, Enshi, China; ^3^Department of Oncology, Laizhou People’s Hospital, Yantai, China; ^4^Department of Oncology, Zhangdian District Traditional Chinese Medicine Hospital, Zibo, China; ^5^Department of Radiotherapy, Tianjin First Central Hospital, Tianjin, China

**Keywords:** hepatocellular carcinoma, Chinese herbal injections, transcatheter arterial chemoembolization, cumulative logit regression, Bayesian network meta-analysis

## Abstract

**Objectives:**

This study evaluated the immunomodulatory effects and clinical efficacy of Chinese herbal injections (CHIs) combined with transcatheter arterial chemoembolization (TACE) in hepatocellular carcinoma (HCC) patients using cumulative logit regression and Bayesian network meta-analysis.

**Methods:**

A systematic review of 48 randomized controlled trials (RCTs) involving 4,293 HCC patients was conducted using PubMed, Cochrane Library, EMBASE, Scopus, and Google Scholar. Outcomes included immune markers (CD3+, CD4+, CD8+, CD4+/CD8+ ratio, and NK cells), clinical response rate, and overall survival (6-month, 1-year, and 2-year). Cumulative logit regression and Bayesian network meta-analysis were applied to synthesize ordinal and continuous outcomes.

**Results:**

Compared to TACE alone, Aidi, Compound Kushen, and Huachansu significantly enhanced the immune function. Aidi increased CD3+ T cells (MD = 10.95, 95% CI: 8.04–13.86), CD4+ T cells (MD = 7.13, 4.37–9.89), CD4+/CD8+ ratio (MD = 0.31, 0.20–0.41), and NK cells (MD = 6.30, 4.49–8.12), while Compound Kushen showed the highest CD4+/CD8+ improvement (MD = 0.47, 0.37–0.56) and NK cell elevation (MD = 9.11, 7.32–10.91). Huachansu increased CD3+ T cells (MD = 8.74, 4.43–13.06) and CD4+ T cells (MD = 8.00, 4.21–11.80). For clinical outcomes, Compound Kushen (HR = 2.57, 1.9–3.59) and Aidi (HR = 2.28, 1.68–3.18) improved clinical response rates versus TACE. Aidi enhanced 6-month (OR = 2.57, 1.44–4.56) and 1-year survival (OR = 2.46, 1.56–3.88), whereas Huachansu (OR = 3.47, 2.42–4.97) and Compound Kushen (OR = 2.91, 1.07–7.89) improved 2-year survival.

**Conclusion:**

Aidi, Compound Kushen, and Huachansu enhance immune function and survival in HCC patients when combined with TACE. Compound Kushen showed the most significant immunomodulatory effects, while Aidi and Huachansu improved short- and long-term survival, respectively. Further high-quality head-to-head RCTs are required to validate these results.

## Introduction

Hepatocellular carcinoma (HCC) represents the predominant histologic subtype of primary liver cancer, accounting for 90% of cases globally, and is projected to surpass 1 million annual diagnoses by 2025 (1). This malignancy poses particular public health challenges in China, where chronic hepatitis B infection drives 55% of worldwide HCC mortality—a burden compounded by late-stage diagnosis and limited therapeutic options for intermediate/advanced disease (2). The socioeconomic ramifications are equally consequential, with treatment costs exceeding 300% of China’s per capita GDP and 5-year survival rates below 12% for unresectable cases (3).

Trans-arterial chemoembolization (TACE) remains the cornerstone of locoregional therapy for intermediate-stage HCC. Recent advancements, including drug-eluting bead TACE (DEB-TACE) and cone-beam CT-guided superselective embolization, have improved tumor targeting and reduced systemic toxicity (4). Despite these innovations, long-term outcomes remain suboptimal, with a single-center study reporting a median progression-free survival of 8.2 months and 58% of patients requiring retreatment within 12 months (5). This limitation stems not only from incomplete tumor necrosis but also from TACE-induced immunogenic stress—a double-edged sword that simultaneously exposes tumor antigens and triggers compensatory immunosuppressive pathways.

Emerging immunological insights reveal a therapeutic paradox: While TACE enhances tumor antigen exposure, compensatory inflammatory responses suppress CD4+/CD8+ T-cell ratios (median reduction of 28%) and impair natural killer (NK) cell cytotoxicity (activity decline of 22–40%)—creating an immunosuppressive milieu facilitating recurrence in 68% of patients within 18 months (6, 7). This underscores the critical need for adjuvant therapies capable of modulating post-TACE immune dysregulation.

Chinese herbal injections (CHIs) represent a unique class of adjuvants deeply rooted in traditional medicine, yet increasingly validated by modern pharmacology. Since 2005, the China Food and Drug Administration has approved 12 CHIs for oncology, with Aidi, Compound Kushen, and Huachansu constituting 68% of HCC-related prescriptions (8, 9). These polypharmacologic agents target multiple oncogenic pathways: Aidi upregulates PD-1/PD-L1 axis inhibitors, Compound Kushen suppresses NF-κB-mediated cytokine storms, and Huachansu inhibits hypoxia-inducible factor 1α (HIF-1α)-driven angiogenesis (10, 11). Despite this mechanistic promise, clinical adoption remains fragmented, with a 2022 national survey revealing that only 34% of tertiary centers utilize standardized CHI protocols alongside TACE (12).

Nevertheless, clinical adoption remains hampered by three critical evidence gaps: heterogeneity in outcome reporting across trials (67% dichotomize ordinal response data), absence of direct comparative efficacy data (93% of RCTs evaluate single-CHI regimens), and insufficient characterization of immune recovery patterns (3, 13, 14). The burgeoning field of immuno-interventional oncology now demands rigorous methodologies to evaluate CHIs-TACE synergism, particularly given the recent failure of checkpoint inhibitor-TACE combinations in phase III trials (e.g., IMbrave050), which highlighted the risks of unmodulated post-embolization inflammation (15).

To address these limitations, we developed an innovative analytical framework integrating cumulative logit regression with Bayesian network meta-analysis—the first application of this dual methodology in CHI research. Traditional approaches systematically underestimate therapeutic effects on intermediate outcomes (partial response/stable disease) by collapsing ordinal scales into binary endpoints, discarding 62% of response hierarchy information according to recent methodological audits (16). Our model advances the field by preserving outcome gradations through proportional odds modeling while enabling probabilistic treatment ranking via Markov chain Monte Carlo simulations. This synergistic approach provides clinicians with three critical innovations: (a) enhanced precision in detecting differential treatment effects across response levels, (b) quantitative evaluation of immune parameter restoration, and (c) economic evaluation of cost-effectiveness ratios.

Our study establishes a precedent in two key dimensions: as the first immune-focused network meta-analysis of CHIs-TACE combinations and the inaugural demonstration of ordinal-outcome Bayesian modeling in herbal medicine research. These methodological advances empower evidence-based decision-making for critical clinical questions regarding optimal CHI selection based on immunological targets, survival benefits, and cost considerations.

## Methods

This meta-analysis was carried out in line with the principles outlined in the “Preferred Reporting Items for Systematic Reviews and Meta-Analyses (PRISMA) guidelines” (17).

### Searching strategy

A comprehensive search was conducted across PubMed, Cochrane Library, Embase, Scopus, and Google Scholar from inception to 3 December 2024, without restrictions on publication year, language, or blinding method. To ensure the feasibility of data extraction and alignment with the primary sources of CHIs-related research, only studies published in English or Chinese were included. The search strategy combined Medical Subject Headings (MeSH) terms and free-text keywords. An example of the PubMed search syntax is provided below:

#1 (Liver Neoplasms) OR (Carcinoma, Hepatocellular) [MeSH Terms].

#2 (Hepatic Neoplasm*) OR (Liver Cancer) OR (Hepatocellular Carcinomas) OR (Hepatoma *) [Title/Abstract].

#3 #1 OR #2.

#4 (Aidi-injection) OR (aidi injection) OR (AD injection) OR (eddy injection) [Title/Abstract].

#5 (Brucea javanica oil emulsion injection) [Title/Abstract].

#6 (Fufang Kushen injection) OR (Compound Sophora injection) OR (Compound Kushen injection) OR (Compound matrine injection) [Title/Abstract].

#7 (Cinobufagin) OR (Cinobufacin injection) OR (cinobufacini injection) OR (Huachansu injection) OR (cinobufotalin injection) [Title/Abstract].

#8 (kang-ai injection) OR (Kangai injection) [Title/Abstract].

#9 (Kanglaite injection) OR (kanglaite) [Title/Abstract].

#10 (Shenqifuzheng injection) [Title/Abstract].

#11 (Xiaoaiping injection) [Title/Abstract].

#12 #4 OR #5 OR #6 OR #7 OR #8 OR #9 OR #10 OR #11.

#13 #3 AND #12.

### Inclusion criteria

The inclusion criteria are as follows:

(I) Randomized controlled trials (RCTs) of CHIs combined with TACE in the treatment of hepatocellular carcinoma;
(II) The CHIs administrated in patients for HCC were authorized by the China Food and Drug Administration;
(III) Patients received exclusive CHIs plus TACE without any other treatment including radiotherapy or surgery;
(IV) Data could be unambiguously extracted or categorized.

### Exclusion criteria

The exclusion criteria are as follows:

(I) The treatment group received CHIs in conjunction with other agents (e.g., chemotherapy and targeted therapy);
(II) Repeated publications of literature or overlapping cohorts;
(III) Studies with incomplete or inaccessible data after two attempts to contact the corresponding authors via email (within a 4-week timeframe);
(IV) Reviews, case reports, single-arm studies, or trials unrelated to the research topic; conference proceedings.

### Data extraction and quality assessment

Two investigators (WJG and YJZ) independently conducted the systematic search and performed dual-phase screening using Covidence software (Version 2.0, Veritas Health Innovation), with conflicts resolved through consensus meetings involving a third reviewer (QFL). Standardized extraction templates were developed *a priori* to capture study characteristics (author, publication year, journal), population demographics (sample size, sex ratio, mean age ± standard deviation), intervention protocols (CHI dosage, administration frequency, TACE technique), and outcome metrics (immune parameters, survival rates, variance estimates). Methodological rigor was assessed using the Cochrane Risk of Bias 2.0 (RoB 2.0) tool, with particular attention to randomization integrity, allocation concealment, blinding procedures, and outcome reporting completeness.

### Outcome set

Primary endpoints focused on cellular immunocompetence, including absolute counts of CD3+ T lymphocytes (cells/μL), CD4+ helper T cells (cells/μL), CD8+ cytotoxic T cells (cells/μL), the CD4+/CD8+ ratio, and natural killer (NK) cell concentrations (CD56+CD16+ cells/μL). Secondary clinical outcomes incorporated modified Response Evaluation Criteria in Solid Tumors (mRECIST) objective response rates and overall survival probabilities at 6-month, 1-year, and 2-year intervals. All immunological measures were standardized to baseline-adjusted changes from pre-treatment values to account for inter-laboratory assay variability.

### Statistical analysis

Network meta-analysis (NMA) was performed to calculate odds ratios (ORs) for dichotomous variables and mean differences (MD) for continuous variables. A random-effects model was applied when heterogeneity exceeded 50% and *p* < 0.01; otherwise, a fixed-effects model was used. Cumulative ranking curves Surface Under the Cumulative Ranking Curve (SUCRAs) were computed to estimate the probability of each treatment being the most effective, with higher SUCRA values indicating a greater likelihood of being the most effective treatment. The importance of effect sizes between treatment pairs was assessed using the net league table, also referred to as the algebraic matrix. To evaluate the robustness of the findings, overall and loop inconsistency tests were conducted. Network funnel plots were generated to detect small sample effects and publication bias. All procedures were carried out using STATA 16.0 MP.

For ordinal data, common logarithm odds ratios (lgOR) and their standard errors (selgOR) were calculated using the cumulative logit regression model in SAS 9.4. These results were then input into R 3.6.3 for Bayesian network meta-analysis, generating hazard ratios (HRs) through matrix-based methods. The use of MD values for continuous outcomes and OR/HR values for dichotomous survival data is based on the specific nature of the outcomes. Trace plots, density plots, and convergence plots were drawn to assess the convergence of the Bayesian model. Statistical significance was determined based on a *p*-value threshold of 0.05.

This approach of combining cumulative logit regression with Bayesian network meta-analysis represents an innovative methodological advance, allowing for more accurate modeling of ordinal outcomes in immunological responses and providing robust, probabilistic treatment rankings. The combination of these methods is particularly valuable in addressing the limitations of traditional approaches, offering improved flexibility and accuracy in assessing treatment effects when direct comparisons are limited.

## Results

### Literature search and study attributes and quality

**Figure 1:**
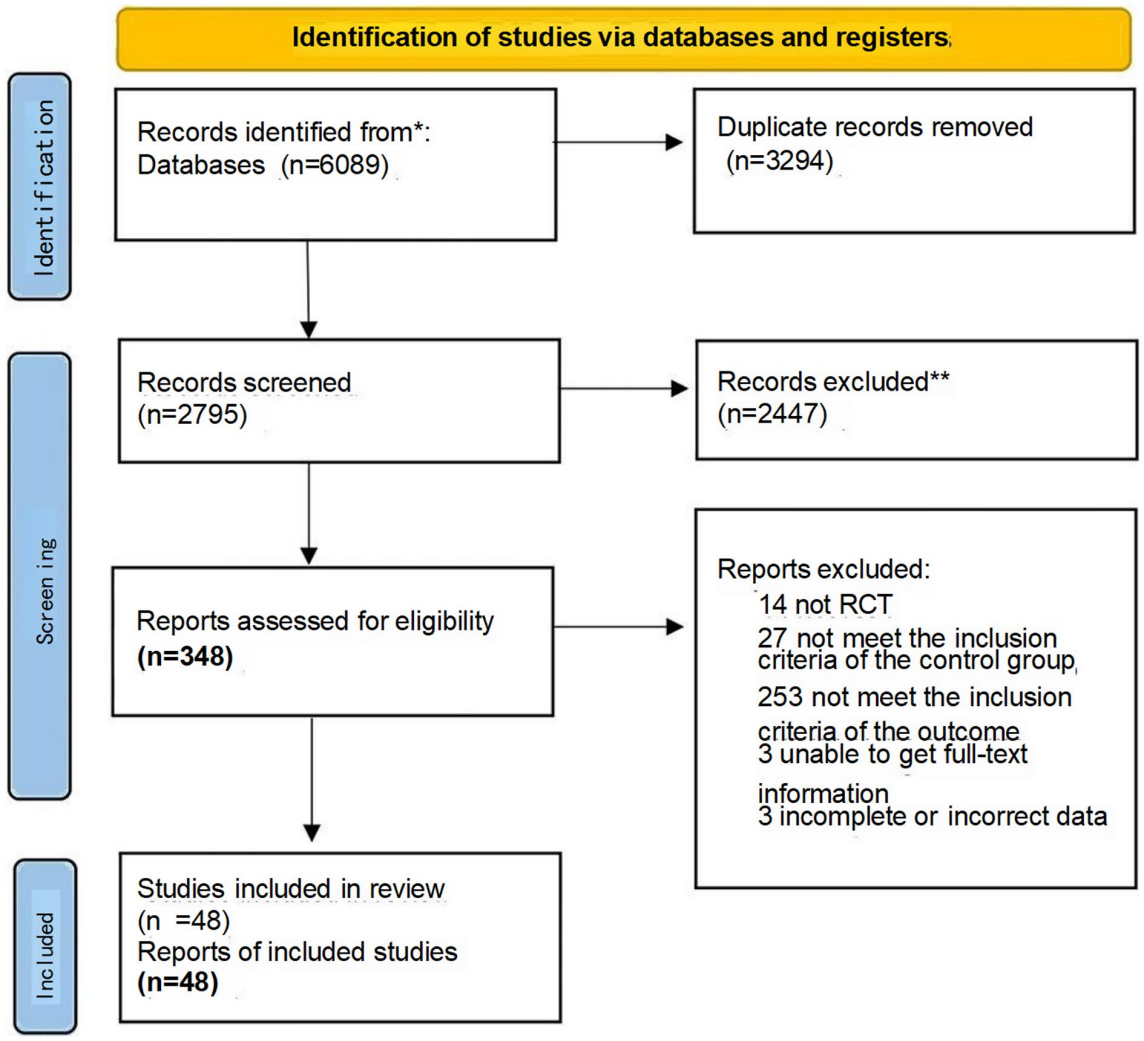
PRISMA flow diagram of the search process for studies.

**Figure 2:**
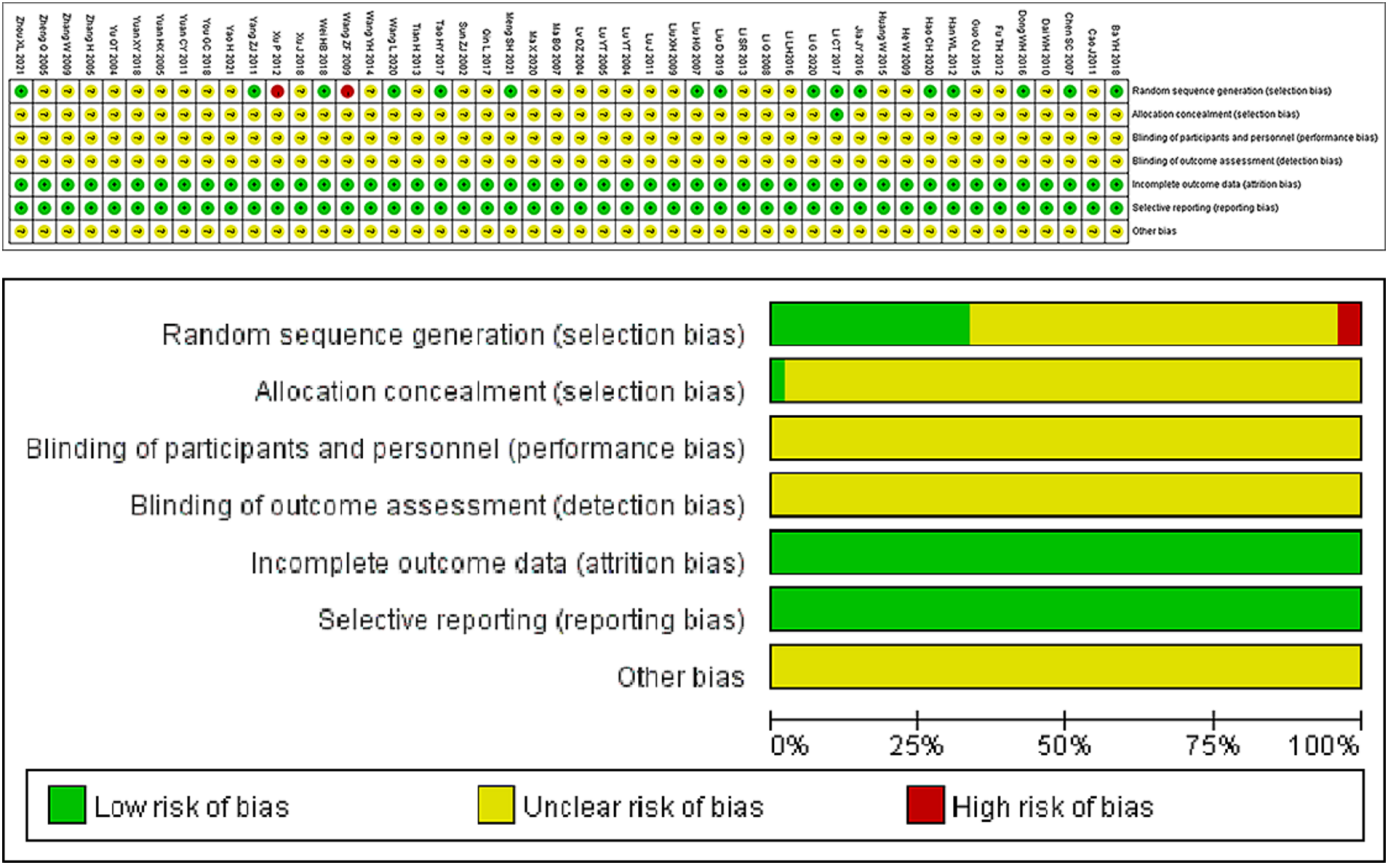
Risk of bias graph.

A total of 6,089 articles were identified from electronic databases, and 48 randomized controlled trials were finally included after screening (Figure 1). Overall, the standard of the included RCTs was suboptimal, with comprehensive data regarding bias risk presented in Figure 2. The 48 RCTs enrolled 4,295 patients; 2,169 of those who took part in the experimental group acquired a combination of CHIs and TACE, and 2,124 patients in the comparison group had only TACE. The average age of patients varied from 36 to 73 years. In all studies, the percentile of male patients was greater than 50%. Details of relevant studies and patients with HCC are presented in the table of baseline characteristics in Supplementary Table S1.

### T-lymphocyte subsets and NK cells

**Figure 3:**
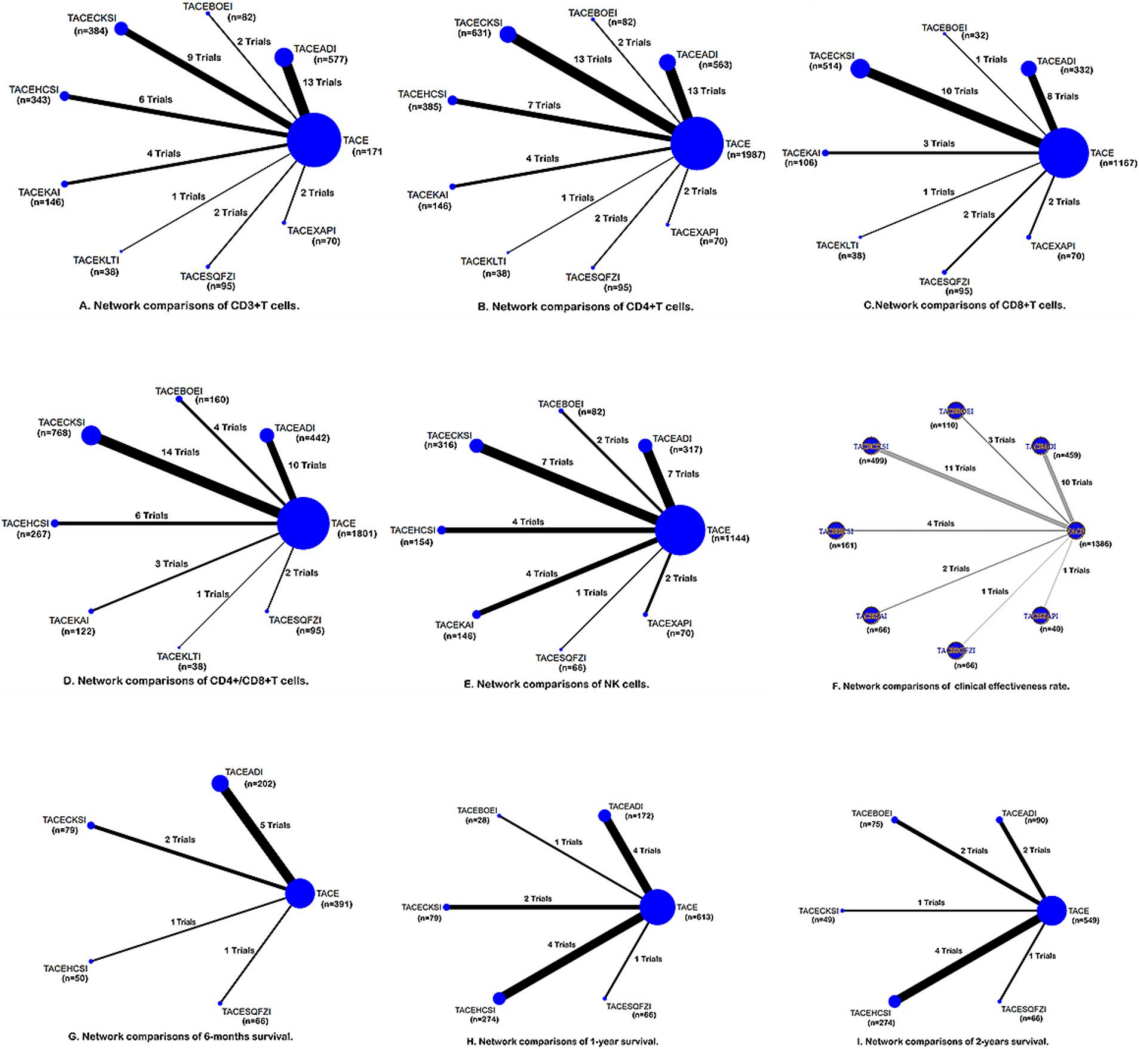
Network graphs of different outcomes: **(A)** CD3^+^; **(B)** CD4^+^; **(C)** CD8^+^; **(D)** CD4^+^/CD8^+^; **(E)** NK; **(F)** clinical response rate; **(G)** 6-month survival; **(H)** 1-year survival; **(I)** 2-year survival. The width of the lines in the network graph is proportional to the number of RCTs used for the comparisons, and the node sizes correspond to the total sample sizes for the treatments. The clinical effective rate graph F was made with R3.6.3. TACEADI, TACE+Aidi injection; TACECKSI, TACE+Compound Kushen injection; TACEBOEI, TACE+ Bruisea Oil Emulsion injection; TACEKAI, TACE+Kangai injection; TACEHCSI, TACE+Huachansu injection; TACEKLTI, TACE+Kanglaite injection; TACESQFZI, TACE+Shenqi Fuzheng injection; TACEXAPI, TACE+Xiaoaiping injection.

Of 48 studies, 39 reported CD3+ T cells involving 8 CHIs (Figure 3A). Compared to TACE, Aidi (MD = 10.95, 95%CI: 8.04–13.86), Compound Kushen (MD = 8.49, 95%CI: 5.04–11.95), Huachansu (MD = 8.74, 95%CI: 4.43–13.06), Kangai (MD = 9.89, 95%CI: 4.58–15.21), Kanglaite (MD = 11.00, 95%CI: 0.76–21.24), Shenqi Fuzheng (MD = 8.17, 95%CI: 0.89–15.46), and Xiaoaiping (MD = 27.54, 95%CI: 19.31–35.77) combined with TACE significantly enhanced the number of CD3+T cells. Xiaoaiping demonstrated significantly greater efficacy compared to the other seven CHIs (Table 1) and ranked the best (SUCRA = 99.9%) (Table 2).

**Table 1:** Statistical results of network meta-analysis for the main outcomes (MD/OR value, 95% CI).

		CD3+	CD4+	CD8+	CD4+/CD8+	NK	6-month survival	1-year survival	2-years survival
TACE	vs								
ADI+TACE		**−10.95 (−13.86,-8.04)**	**−7.13 (−9.89,-4.37)**	−0.82 (−4.75,3.11)	**−0.31 (−0.41,-0.20)**	**−6.30 (−8.12,-4.49)**	**0.39 (0.22,0.69)**	**0.41 (0.26,0.64)**	**0.51 (0.26,1.03)**
BOEI+TACE		−5.48 (−12.73,1.78)	**−7.88 (−14.86,-0.90)**	−5.34 (−16.32,5.64)	**−0.27 (−0.45,-0.09)**	**−5.44 (−9.02,-1.86)**	-	0.48 (0.04,5.64)	**0.95 (0.45,2.00)**
CKSI+TACE		**−8.49 (−11.95,-5.04)**	**−8.85 (−11.59,-6.12)**	−0.43 (−3.93,3.07)	**−0.47 (−0.56,-0.37)**	**−9.11 (−10.91,-7.32)**	0.38 (0.12,1.23)	0.56 (0.25,1.26)	**0.34 (0.13,0.93)**
HCSI+TACE		**−8.74 (−13.06,-4.43)**	**−8.00 (−11.80,-4.21)**	-	**−0.27 (−0.41,-0.12)**	**−6.48 (−9.02,-3.94)**	0.89 (0.35,2.26)	**0.30 (0.21,0.43)**	**0.29 (0.20,0.41)**
KAI+TACE		**−9.89 (−15.21,-4.58)**	−4.77 (−9.82,0.27)	−1.52 (−7.91,4.86)	−0.09 (−0.34,0.15)	**−8.72 (−11.22,-6.23)**	-	-	-
KLTI+TACE		**−11.00 (−21.24,-0.76)**	**−11.00 (−20.84,-1.16)**	−1.00 (−11.95,9.95)	−0.22 (−0.55,0.11)	-	-	-	-
SQFZI+TACE		**−8.17 (−15.46,-0.89)**	−5.48 (−12.46,1.50)	−2.40 (−10.21,5.40)	**−0.32 (−0.60,-0.04)**	**−5.00 (−9.97,-0.03)**	**0.11 (0.01,0.92)**	**0.08 (0.03,0.21)**	**0.16 (0.06,0.47)**
XAPI+TACE		**−27.54 (−35.77,-19.31)**	**−16.11 (−23.73,-8.48)**	−3.71 (−11.57,4.14)	-	-	-	-	-
ADI+TACE	vs								
BOEI+TACE		−5.47 (−13.29,2.34)	−0.75 (−8.26,6.75)	−4.52 (−16.18,7.14)	−0.03 (−0.25,0.18)	−0.87 (−4.88,3.15)	-	0.84 (0.07,10.30)	0.54 (0.20,1.50)
CKSI+TACE		−2.46 (−6.98,2.06)	−1.73 (−5.61,2.16)	−1.25 (−6.51,4.01)	**−0.16 (−0.30,-0.02)**	**−2.81 (−5.37,-0.26)**	1.03 (0.28,3.81)	0.72 (0.29,1.82)	0.67 (0.20,2.26)
HCSI+TACE		−2.21 (−7.41,2.99)	−0.88 (−5.57,3.81)	-	−0.04 (−0.22,0.14)	−0.18 (−3.31,2.94)	0.35 (0.12,1.04)	0.73 (0.41,1.32)	0.56 (0.26,1.22)
KAI+TACE		−1.06 (−7.11,5.00)	−2.36 (−8.10,3.39)	−2.34 (−9.84,5.16)	−0.21 (−0.48,0.05)	−2.42 (−5.51,0.67)	-	-	-
KLTI+TACE		0.05 (−10.59,10.69)	−3.87 (−14.10,6.35)	−1.82 (−13.45,9.81)	−0.09 (−0.43,0.26)	-	-	-	-
SQFZI+TACE		−2.78 (−10.62,5.07)	−1.65 (−9.16,5.86)	−1.59 (−10.32,7.15)	−0.01 (−0.31,0.29)	−1.30 (−6.60,3.99)	0.29 (0.03,2.55)	**0.20 (0.07,0.57)**	0.32 (0.09,1.12)
XAPI+TACE		**−16.59 (−25.32,-7.87)**	**−8.98 (−17.09,-0.87)**	−2.90 (−11.68,5.89)	-	**−12.19 (−17.38,-7.01)**	-	-	-
BOEI+TACE	vs								
CKSI+TACE		−3.02 (−11.05,5.02)	−0.97 (−8.47,6.52)	−5.77 (−17.29,5.75)	−0.19 (−0.40,0.01)	−3.68 (−7.68,0.32)	-	0.85 (0.06,11.37)	0.36 (0.10,1.26)
HCSI+TACE		−3.27 (−11.71,5.17)	−0.13 (−8.07,7.82)	-	−0.01 (−0.24,0.23)	−1.05 (−5.44,3.34)	-	0.62 (0.05,7.46)	**0.30 (0.13,0.70)**
KAI+TACE		−4.42 (−13.41,4.57)	−3.11 (−11.72,5.50)	−6.86 (−19.56,5.84)	−0.18 (−0.48,0.12)	−3.29 (−7.65,1.07)	-	-	-
KLTI+TACE		−5.52 (−18.07,7.02)	−3.12 (−15.19,8.94)	−6.34 (−21.84,9.16)	−0.05 (−0.42,0.32)	-	-	-	-
SQFZI+TACE		−2.70 (−12.98,7.58)	−2.40 (−12.27,7.47)	−2.94 (−16.40,10.53)	−0.05 (−0.38,0.29)	−0.44 (−6.56,5.69)	-	0.17 (0.01,2.36)	**0.17 (0.05,0.63)**
XAPI+TACE		**−22.07 (−33.04,-11.10)**	−8.23 (−18.56,2.11)	−1.63 (−15.12,11.87)	-	**−13.06 (−19.08,-7.03)**	-	-	-
CKSI+TACE	vs								
HCSI+TACE		−0.25 (−5.78,5.28)	−0.85 (−5.53,3.83)	-	**−0.20 (−0.37,-0.03)**	−2.63 (−5.74,0.48)		0.53 (0.22,1.28)	0.84 (0.29,2.42)
KAI+TACE		−1.40 (−7.74,4.94)	−4.08 (−9.82,1.65)	−1.09 (−8.37,6.19)	**−0.37 (−0.64,-0.11)**	−0.39 (−3.46,2.68)	-	-	-
KLTI+TACE		−2.51 (−13.31,8.30)	−2.15 (−12.36,8.07)	0.57 (−10.92,12.06)	−0.25 (−0.58,0.09)	-	-	-	-
SQFZI+TACE		−0.57 (−9.04,7.90)	−3.38 (−10.87,4.12)	−2.84 (−11.39,5.72)	−0.15 (−0.44,0.15)	−4.11 (−9.40,1.17)		**0.15 (0.04,0.50)**	0.48 (0.11,2.02)
XAPI+TACE		**−18.80 (−28.09,-9.51)**	−7.25 (−15.35,0.85)	−4.15 (−12.75,4.46)	-	**−9.38 (−14.55,-4.21)**	-	-	-
HCSI+TACE	vs								
KAI+TACE		−1.15 (−7.99,5.69)	−3.23 (−9.55,3.08)	-	−0.17 (−0.46,0.11)	−2.24 (−5.80,1.32)	-	-	-
KLTI+TACE		−2.26 (−13.36,8.85)	−3.00 (−13.55,7.55)	-	−0.05 (−0.40,0.31)	-	-	-	-
SQFZI+TACE		−0.57 (−9.04,7.90)	−2.53 (−10.47,5.42)	-	−0.05 (−0.37,0.26)	−1.48 (−7.07,4.10)		**0.28 (0.10,0.74)**	0.57 (0.19,1.72)
XAPI+TACE		**−18.80 (−28.09,-9.51)**	−8.10 (−16.62,0.41)	-	-	**−12.01 (−17.49,-6.53)**	-	-	-
KAI+TACE	vs								
KLTI+TACE		−1.11 (−12.64,10.43)	−6.23 (−17.29,4.83)	−0.52 (−13.19,12.15)	−0.13 (−0.53,0.28)	-	-	-	-
SQFZI+TACE		−1.72 (−10.74,7.30)	−0.71 (−9.32,7.91)	−3.93 (−14.01,6.16)	−0.23 (−0.60,0.15)	−3.72 (−9.29,1.84)	-	-	-
XAPI+TACE		**−17.65 (−27.44,-7.86)**	**−11.34 (−20.48,-2.19)**	−5.24 (−15.36,4.89)	-	**−9.77 (−15.23,-4.32)**	-	-	-
KLTI+TACE	vs								
SQFZI+TACE		−2.83 (−15.39,9.74)	−5.52 (−17.59,6.54)	−3.40 (−16.85,10.04)	−0.10 (−0.53,0.33)	-	-	-	-
XAPI+TACE		**−16.54 (−29.67,-3.41)**	−5.11 (−17.56,7.34)	−4.71 (−18.19,8.76)	-	-	-	-	-
SQFZI+TACE	**vs**								
XAPI+TACE		**−19.37 (−30.36,-8.38)**	**−10.63 (−20.97,-0.29)**	−1.31 (−12.38,9.76)	-	**−13.50 (−20.45,-6.55)**	-	-	-

Bold results indicate statistically significant differences between groups; ADI, Aidi injection; CKSI, Fufang Kushen injection; BOEI, Brucea javanica oil emulsion injection; KAI, Kangai injection; HCSI, Huachansu injection; KLTI, Kanglaite injection; SQFZI, Shenqi Fuzheng injection; XAPI, Xiaoaiping injection.

**Table 2:** Surface under the cumulative ranking probability (SUCRA) results of main outcomes.

Intervention	CD3^+^		CD4^+^		CD8^+^		CD4^+^/CD8^+^		NK		6-month survival		1-year survival		2-year survival	
	SUCRA (%)	Rank	SUCRA (%)	Rank	SUCRA (%)	Rank	SUCRA (%)	Rank	SUCRA (%)	Rank	SUCRA (%)	Rank	SUCRA (%)	Rank	SUCRA (%)	Rank
TACE	85.3	1	65.4	3	68.1	2	60.7	4	-	-	16.8	4	7.2	6	9.8	6
ADI+TACE	51	5	71	2	88.6	1	39.8	5	33.1	5	64.7	2	49.2	3	44.4	4
BOEI+TACE	9.1	8	13.8	8	54.1	4	22.5	6	-	-	-	-	43.6	4	14.9	5
CKSI+TACE	80.4	2	59.3	4	-	-	91.4	1	-	-	63.6	3	33.7	5	63.7	3
HCSI+TACE	46.6	6	87.6	1	47.5	5	84.4	2	66.4	3	13.5	5	68.4	2	74.4	2
KAI+TACE	73.9	3	52.2	6	-	-	-	-	83.3	2	-	-	-	-	-	-
KLTI+ TACE	30.8	7	34.3	7	31.3	6	7.2	8	50.1	4	-	-	-	-	-	-
SQFZI+TACE	65.5	4	58.4	5	6.3	7	77.6	3	-		91.5	1	97.9	1	92.8	1
XAPI+TACE	-	-	-	-	-	-	-	-	16.4	6	-	-	-	-	-	-

ADI, Aidi injection; CKSI, Fufang Kushen injection; BOEI, Brucea javanica oil emulsion injection; KAI, Kangai injection; HCSI, Huachansu injection; KLTI, Kanglaite injection; SQFZI, Shenqi Fuzheng injection; XAPI, Xiaoaiping injection.

Of 48 studies, 44 reported CD4+ T cells involving 8 CHIs (Figure 3B). Compared to TACE, Aidi (MD = 7.13, 95% Cl: 4.37–9.89), Brucea javanica oil emulsion (MD = 7.88, 95%CI: 0.90–14.86), Compound Kushen (MD = 8.85, 95%CI: 6.12–11.59), Huachansu (MD = 8.00, 95%CI: 4.21–11.80), Kanglaite (MD = 11.00, 95%CI: 1.16–20.84), and Xiaoaiping (MD = 16.11, 95%CI: 8.48–23.73) combined with TACE observably boost CD4+ T-cell number. Xiaoaiping was superior to Aidi, Shenqi Fuzheng, and Kangai (Table 1).

Of 48 studies, 27 reported CD8+T cells involving 7 CHIs (Figure 3C). No statistically significant difference among those regimens and control was observed for increasing CD8+ T-cell numbers (Table 1).

Of 48 studies, 39 reported CD4+/CD8+ cells ratio involving 7 CHIs (Figure 3D). Compared to TACE, Aidi (MD = 0.31, 95%CI: 0.20–0.41), Brucea javanica oil emulsion (MD = 0.27, 95%CI: 0.09–0.45), Compound Kushen (MD = 0.47, 95%CI: 0.37–0.56), Huachansu (MD = 0.27, 95%CI: 0.12–0.41), and Shenqi Fuzheng (MD = 0.32, 95%CI: 0.04–0.60) significantly improved the CD4+/CD8+ cell ratio. Compound Kushen was superior to Kangai and Huachansu (Table 1).

Of 48 studies, 27 reported NK cells involving 7 CHIs (Figure 3E). Compared to TACE, Aidi (MD = 6.30, 95%CI: 4.49–8.12), Brucea javanica oil emulsion (MD = 5.44, 95%CI: 1.86–9.02), Compound Kushen (MD = 9.11, 95%CI: 7.32–10.91), Huachansu (MD = 6.48, 95%CI: 3.94–9.02), Kangai (MD = 8.72, 95%CI: 6.23–11.22), Shenqi Fuzheng (MD = 5.00, 95%CI: 0.03–9.97), and Xiaoaiping (MD = 18.50, 95%CI: 13.64–23.35) dramatically increased NK cell levels. Xiaoaiping was considerably superior to the rest of the 7 CHIs (Table 1) and ranked the best (SUCRA = 100.0%) (Table 2). Compound Kushen was superior to Aidi (Table 1).

### Clinical response rate

Of 48 studies, 32 reported a clinical response rate involving 7 CHIs (Figure 3F).

Compared to TACE, Aidi (HR = 2.28, 95%CI: 1.68–3.18), Brucea javanica oil emulsion (HR = 3.22, 95%CI: 1.71–6.35), Compound Kushen (HR = 2.57, 95%CI: 1.9–3.59), and Huachansu (HR = 1.76, 95%CI: 1.03–3) combined with TACE significantly improved the clinical response rate (Table 3).

**Table 3:** Results of the network meta-analysis of the clinical response rate.

	TACE	ADI+TACE	BOEI+TACE	CKSI+TACE	HCSI+TACE	KAI+TACE	SQFZI+TACE	XAPI+TACE
TACE	1	**2.28 (1.68, 3.18)**	**3.22 (1.71, 6.35)**	**2.57 (1.9, 3.59)**	**1.76 (1.03, 3)**	1.33 (0.62, 2.88)	1.87 (0.77, 4.58)	2.14 (0.79, 5.79)
ADI+TACE	0.44 (0.31, 0.6)	1	1.41 (0.69, 2.96)	1.13 (0.72, 1.76)	0.77 (0.41, 1.42)	0.58 (0.25, 1.33)	0.82 (0.32, 2.1)	0.94 (0.32, 2.63)
BOEI+TACE	0.31 (0.16, 0.58)	0.71 (0.34, 1.45)	1	0.8 (0.39, 1.63)	0.55 (0.23, 1.24)	0.41 (0.15, 1.11)	0.58 (0.19, 1.71)	0.66 (0.2, 2.13)
CKSI+TACE	0.39 (0.28, 0.53)	0.89 (0.57, 1.38)	1.25 (0.61, 2.59)	1	0.68 (0.36, 1.26)	0.51 (0.22, 1.19)	0.73 (0.28, 1.86)	0.83 (0.29, 2.33)
HCSI+TACE	0.57 (0.33, 0.97)	1.3 (0.7, 2.45)	1.83 (0.81, 4.32)	1.46 (0.79, 2.78)	1	0.76 (0.3, 1.94)	1.06 (0.38, 3.03)	1.22 (0.39, 3.76)
KAI+TACE	0.75 (0.35, 1.6)	1.72 (0.75, 3.95)	2.43 (0.9, 6.67)	1.94 (0.84, 4.47)	1.32 (0.52, 3.34)	1	1.41 (0.44, 4.56)	1.6 (0.46, 5.66)
SQFZI+TACE	0.54 (0.22, 1.3)	1.22 (0.48, 3.15)	1.71 (0.59, 5.33)	1.38 (0.54, 3.6)	0.94 (0.33, 2.64)	0.71 (0.22, 2.28)	1	1.15 (0.3, 4.36)
XAPI+TACE	0.47 (0.17, 1.26)	1.07 (0.38, 3.1)	1.5 (0.47, 5.07)	1.2 (0.43, 3.48)	0.82 (0.27, 2.54)	0.62 (0.18, 2.17)	0.87 (0.23, 3.31)	1

ADI, Aidi injection; CKSI, Fufang Kushen injection; BOEI, Brucea javanica oil emulsion injection; KAI, Kangai injection; HCSI, Huachansu injection; KLTI, Kanglaite injection; SQFZI, Shenqi Fuzheng injection; XAPI, Xiaoaiping injection. The bold values indicate they are statistically significant.

### Overall survival

Of 48 studies, 9 reported 6-month survival, involving 4 CHIs (Figure 3G). Compared to TACE, Aidi (OR = 2.57, 95%CI: 1.44–4.56), Shenqi Fuzheng (OR = 8.97, 95%CI: 1.09–73.86) combined with TACE prominently increased the survival of patients at 6 months (Table 1).

Of 48 studies, 12 reported 1-year survival, involving 5 CHIs (Figure 3H). Compared to TACE, Aidi (OR = 2.46, 95%CI: 1.56–3.88), Huachansu (OR = 3.35, 95%CI: 2.33–4.82), and Shenqi Fuzheng (OR = 12.17, 95%CI: 4.83–30.69) combined with TACE significantly increased the 1-year survival of patients. Shenqi Fuzheng combined with TACE was considerably superior to the rest of the 3 CHIs (Table 1) and ranked the best (SUCRA = 97.9%) (Table 2).

Of 48 studies, 10 reported 2-year survival, involving 5 CHIs (Figure 3I). Compared to TACE, Compound Kushen (OR = 2.91, 95%CI: 1.07–7.89), Huachansu (OR = 3.47, 95%CI: 2.42–4.97), and Shenqi Fuzheng (OR = 6.10, 95%CI: 2.14–17.35) combined with TACE significantly increased the 2-year survival of patients. Shenqi Fuzheng was superior to Compound Kushen (Table 1).

### Small sample effect, inconsistency, and convergency

**Figure 4:**
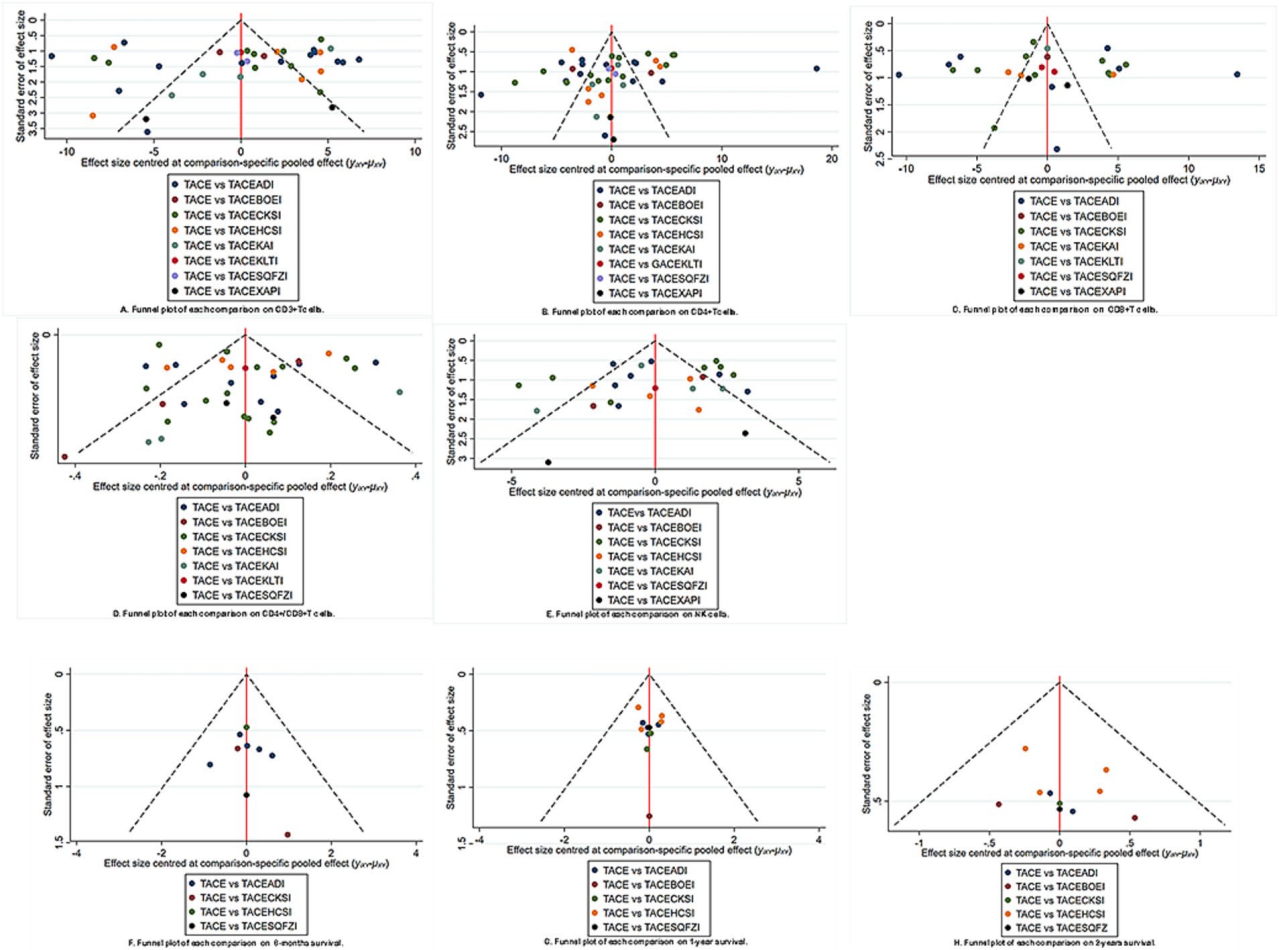
Funnel plot of pairwise comparison among each treatment on **(A)** CD3^+^; **(B)** CD4^+^; **(C)** CD8^+^; **(D)** CD4^+^/CD8^+^; **(E)** NK; **(F)** 6-month survival; **(G)** 1-year survival; **(H)** 2-year survival. Note: TACEADI, TACE+Aidi injection; TACECKSI, TACE+ Compound Kushen injection; TACEBOEI, TACE+ Bruisea Oil Emulsion injection; TACEKAI, TACE+Kangai injection; TACEHCSI, TACE+Huachansu injection; TACEKLTI, TACE+Kanglaite injection; TACESQFZI, TACE+Shenqi Fuzheng injection; TACEXAPI, TACE+Xiaoaiping injection.

The network funnel plots suggest the presence of small sample effects between TACE and TACE+Xiaoaiping, TACE and TACE+Kanglaite, and TACE and TACE+Shenqi Fuzheng in peripheral blood T lymphocyte subsets and overall survival outcomes (Figure 4). The overall and loop inconsistency test found no consistency in any outcome. Trajectory density plots and convergence plots are available in Supplementary materials.

## Discussion

To our knowledge, this is the first network meta-analysis to evaluate the immune effects of CHIs combined with TACE in HCC patients, implementing a cumulative logit regression model for ordinal data in conjunction with Bayesian network meta-analysis using lgOR and selgOR, representing the first study of its kind. It should be noted that the majority of studies utilized chi-square tests and merged the number of patients exhibiting complete response, partial response, and stable disease as a whole to conduct network meta-analysis for binary variants. However, both analytical methodologies are erroneous. First, for ordinal data, the chi-square test is not appropriate; instead, the rank-sum test is the correct approach. Second, again for ordinal data, pooling the three graded levels of efficacy does not make full use of the hierarchy, as it omits the differences among those levels.

There may be wide suspicion regarding the motivation of setting the immunity change as the predominant outcome instead of overall survival in our study. Here are the reasons:

HCC patients experience varying levels of cellular immune function suppression, and changes in T lymphocyte subsets are related to the malignancy of HCC (18). Studies have shown that observing variations in peripheral blood T lymphocyte subset indicators offers a more accurate understanding of the immune condition of cancer patients at a given time, which is important for determining appropriate treatment plans, observing efficacy, and evaluating prognosis (19). Currently, TACE treatment alone often fails to achieve significant therapeutic effects and has a poor effect on immune function restoration (20). Reducing the incidence of low immune function caused by TACE may be the key to preventing and diminishing the invasion and metastasis of tumor cells in patients with HCC after TACE (21). Multiple studies have confirmed that strengthening cellular immunity in HCC patients can extend lifespan (22). Therefore, improving the immune capability of HCC patients was considered a prerequisite for improving OS and PFS.

A network meta-analysis found that patients who received CHIs had a significantly enhanced immune effect (23, 24). Xiaoaiping was the most powerful CHI in salvaging the decline of CD3+T and NK cells. Xiaoaiping injection was an extract of the Marsdenia tenacissima (Roxb.), there were mainly “polysaccharides, C-21 steroidal saponins, organic acids, and alkaloids in Xiaoaiping injection to clear away heat, detoxify, dissolve phlegm, and soften firmness” (25). Its active ingredients can improve the body’s immune function, remarkably inhibiting digestive tract tumors, effectively scavenging oxygen free radicals, and augmenting the surgical efficacy of patients (26). Consistent with the results of this study, immune-related indicators could be surged. Aidi, Compound Kushen, and Huachansu combined with TACE can evidently increase “CD3+T, CD4+T, CD4+/CD8+ cell ratio,” and NK cell level simultaneously. Many fundamental studies have demonstrated that in treating HCC, Aidi, Compound Kushen, and Huachansu could play a certain function in controlling the immune function of patients after TACE (27–30). Compound Kushen injection has significant anti-tumor activity, can effectively interfere with the proliferation of He3B, Hep2, and other liver cancer cells, and can exert its anti-tumor effect by promoting cell differentiation and apoptosis, inhibiting cell metastasis and invasion, and enhancing body immunity (31). It was extracted through standardized Good Manufacturing Practices (GMPs) “from the roots of the medical herbs Kushen (*Radix Sophorae flavescentis*) and Baituling (*Rhizoma Smilacis glabrae*)” (32). Aidi injection has a suppressive impact on the growth of transplanted tumors, and it is believed that it may work through mechanisms such as improving the body’s immunity (33). Aidi injection contains *Ginseng Radix et Rhizoma, Astragali Radix, Acanthopanacis Senticosi Radix Et Rhizoma Seu Caulis*, and *Mylabris* (34). Research has shown that the combined application of Aidi injection and the chemotherapy drug 5-fluorouracil can enhance human immune function and improve anti-stress ability (35). Huachansu injection is a preparation form of Cinobufacin made from Cinobufacin (*Bufo gargarizans Cantor*) extract liquid (36). Studies have shown that Bufadienolides represent the most potent components in Huachansu liver cancer treatment injection (37). Huachansu injection mainly plays a cancer-fighting effect by inhibiting the proliferation and differentiation ability of cancer cells, causing cell apoptosis, and enhancing the immune response of the body’s ability (38).

The high costs of treating HCC impose substantial burdens on family members and society. A report demonstrated that the average injection cost for patients using Compound Kushen injection was 7,086 RMB, the cost-effectiveness ratio determined based on the remission rate was 12,618, and the cost-effectiveness ratio derived from the effective rate was 9,084; this cost is easy to be accepted by patients with HCC (39). Another study showed that the cost-effectiveness ratio of Aidi was significantly lower than that of Kanglaite and Xiaoaiping, and it is a more economical solution for advanced HCC patients (40). The latest study found that the per capita quality-adjusted life years (QALY) of Aidi combined with TACE in the management of HCC increased by 0.19, the average medical expenditure increased by 10,403RMB, and the incremental cost-effectiveness ratio (ICER) was 54,753, which was less than 1 times the domestic per capita gross domestic product (GDP), indicating that the Aidi treatment plan has cost-effectiveness advantage (41). In previous studies, we found that Aidi combined with TACE ranked third among 19 injections in terms of clinical benefit. Aidi has a better recent benefit rate than Compound Kushen and Huachansu (42).

Based on the analysis of the above results, although there is no significant difference in the efficacy of the three injections of Aidi, Compound Kushen, and Huachansu, there seems to be a trend that Compound Kushen has an advantage over Aidi and Huachansu in improving immunity. Aidi has an advantage in short-term survival, while Compound Kushen and Huachansu are more advantageous in long-term survival. In terms of pharmacoeconomics, it seems that Aidi and Compound Kushen are more acceptable to patients.

### Limitations

First, the methodological quality of the included RCTs was suboptimal. Critical risk-of-bias domains—specifically, blinding of participants, personnel, and outcome assessors—were unreported in all studies, and allocation concealment was similarly absent. These omissions may introduce performance and detection bias, potentially inflating treatment effect estimates for immune markers (e.g., CD4+/CD8+ ratio) and survival outcomes. For example, unblinded assessments of subjective endpoints such as the clinical response rate could systematically favor experimental groups, overstating therapeutic benefits. Second, the evidence base for certain CHIs (e.g., Kanglaite and Xiaoaiping) were limited to 2–3 trials with small sample sizes (n < 100 per arm), reducing the precision of effect estimates and limiting subgroup analyses. This small sample size also makes it difficult to generalize the results to a broader patient population. Third, the absence of head-to-head comparisons among CHIs precludes definitive conclusions about their comparative efficacy. While Bayesian network meta-analysis allows indirect comparisons, these results remain hypothesis-generating and require validation in dedicated RCTs.

## Conclusion

CHIs, particularly Aidi, Compound Kushen, and Huachansu, were promising in augmenting immune function and improving clinical outcomes in HCC patients. Compound Kushen demonstrated potentially superior immunomodulatory effects, while Aidi and Huachansu were more effective in optimizing short- and long-term survival, respectively. Given the methodological limitations of the included studies, such as small sample sizes and lack of blinding, the results should be interpreted with caution. Despite these promising findings, additional rigorous, high-quality direct randomized controlled trials are required to validate these preliminary results and confirm the relative efficacy of the different CHIs.

## Data Availability

The original contributions presented in the study are included in the article/Supplementary material, further inquiries can be directed to the corresponding author.
