# Supplementary material for: Immune regulation and clinical response of Chinese herbal injections combined with TACE in hepatocellular carcinoma: a cumulative logit regression and Bayesian network meta-analysis
Source: Front Med (Lausanne). 2025 Apr 4;12:1567137. doi: 10.3389/fmed.2025.1567137 (PMC12006906; doi:10.3389/fmed.2025.1567137)
Supplement: Supplementary file 1 [file Table_1.doc]

**Table S1. Basic Characteristics of the Included Studies.**

| **Study ID** | Sex（M/F） | Cases（E/C） | Average age (E/C) | KPS score(E/C) | Treatment group intervention | Control group intervention | Outcomes |  |
| --- | --- | --- | --- | --- | --- | --- | --- | --- |
| **Lu YT 2004** | 51/11 | 32/30 | 48.25/47.15 | 30-60 | TACE+ADI | TACE | ①②③④⑤⑥ |  |
| **Yuan HX 2005** | 58/15 | 35/38 | 56/53 | >50 | TACE+ADI | TACE | ①②③④⑥ |  |
| **Zhang H 2005** | 46/4 | 27/23 | 52.4±5.3/52.6±5.4 | >60 | TACE+ADI | TACE | ①②④⑤ |  |
| **Zheng Q 2005** | NR | 48/48 | 51.4/49.8 | >60 | TACE+ADI | TACE | ①②④⑤⑦⑧⑨ |  |
| **Chen SC 2007** | 41/19 | 32/28 | 37-70/36-67 | >60 | TACE+ADI | TACE | ①②③④⑥ |  |
| **Ma BQ 2007** | 78/42 | 60/60 | 44 | ≥60 | TACE+ADI | TACE | ①②④⑥ |  |
| **Dai WH 2010** | 95/5 | 50/50 | 52 | NR | TACE+ADI | TACE | ①②③⑤⑦⑧ |  |
| **Yang ZJ 2011** | 53/7 | 30/30 | 49.8/49 | ≥70 | TACE+ADIl | TACE | ①②③④⑦ |  |
| **Huang W 2015** | 67/49 | 58/58 | 58.1±9.6/55.4±8.1 | NR | TACE+ADI | TACE | ①②④⑤⑥ |  |
| **Qin L 2017** | 39/23 | 32/30 | 44/45.5 | NR | TACE+ADI | TACE | ①②③④⑥⑦⑧ |  |
| **Tao HY 2018** | 77/51 | 64/64 | 59.1±11.2/60.8±12.4 | 55.4 ± 10.1/57.5 ± 9. 2 | TACE+ADI | TACE | ①②③④⑤⑥ |  |
| **Wei HB 2018** | 63/21 | 42/42 | 47.01±11.21/48.03±10.34 | ≥50 | TACE+ADI | TACE | ①②③④⑤⑥⑦⑧⑨ |  |
| **Liu D 2019** | 69/47 | 58/58 | 59.16±12.35/60.45±13.27 | NR | TACE+ADI | TACE | ①②③④⑥ |  |
| **Ma X 2020** | 61/31 | 46/46 | 50.31±6.04/49.68±5.27 | NR | TACE+ADI | TACE | ①④⑥ |  |
| **Zhang W 2009** | 39/15 | 29/25 | 52/51 | NR | TACE+SQFZI | TACE | ①②③④ |  |
| **Xu J 2018** | 79/53 | 66/66 | 48.25±4.5/47.35±4.83 | NR | TACE+SQFZI | TACE | ①②③④⑤⑥⑦⑧⑨ |  |
| **Wang ZF 2009** | 51/32 | 42/41 | 53 | NR | TACE+CKSI | TACE | ①②③⑤⑥ |  |
| **Cao J 2011** | NR | 30/30/30 | NR | NR | TACE+CKSI | TACE | ①②③⑤⑥ |  |
| **Lu J 2011** | 47/27 | 39/35 | 63.4/62.8 | <70;70-80:>80 | TACE+CKSI | TACE | ①②③④⑤ |  |
| **Han WL 2012** | 37/23 | 30/30 | 56/57 | NR | TACE+CKSI | TACE | ①②③④⑥ |  |
| **Xu P 2012** | 38/22 | 30/30 | 53 | NR | TACE+CKSI | TACE | ①②④⑤⑥⑦⑧ |  |
| **Guo GJ 2015** | 92/64 | 78/78 | 46.1±3.2/47.3±2.9 | >70 | TACE+CKSI | TACE | ①②③④⑤⑥ |  |
| **Dong WH 2016** | 137/79 | 108/108 | 56 | NR | TACE+CKSI | TACE | ①②③④⑤⑥ |  |
| **Ba YH 2018** | 48/36 | 42/42 | 56. 8±3. 1/56. 3 ±2. 9 | ≥60 | TACE+CKSI | TACE | ②③④⑥ |  |
| **You GC 2018** | 55/35 | 45/45 | 61.56±2.54/61.92±2.25 | NR | TACE+CKSI | TACE | ②③④⑥ |  |
| **Yuan XY 2018** | 48/42 | 45/45 | 60. 41±8. 43/60. 84 ± 8. 67 | ≥60 | TACE+CKSI | TACE | ①②③ |  |
| **Hao CH 2020** | 60/38 | 49/49 | 57.01 ± 6.82/56.25 ± 6.21 | NR | TACE+CKSI | TACE | ②③④⑤⑥⑦⑧⑨ |  |
| **Li G 2020** | 38/26 | 33/31 | 61.38±6.85/60.74± 7.29 | NR | TACE+CKSI | TACE | ②③⑥ |  |
| **Yao H 2021** | 48/48 | 48/48 | 63.24±2.57/63.58±2.96 | NR | TACE+CKSI | TACE | ①②③④⑤⑥ |  |
| **Zhou XL 2021** | 52/32 | 42/42 | 58.44±4.62/57.62±4.71 | ≥60 | TACE+CKSI | TACE | ①②④⑥ |  |
| **Sun ZJ 2002** | 197/39 | 118/118 | 51.4 | NR | TACE+HCSI | TACE | ①②④⑤⑧⑨ |  |
| **Yu QT 2004** | 102/26 | 64/64 | 45.4 | NR | TACE+HCSI | TACE | ①②③④⑧⑨ |  |
| **Li Q 2008** | 84/12 | 50/46 | 50.2 | ≥60 | TACE+HCSI | TACE | ①②④⑤⑥⑦⑧⑨ |  |
| **Liu XH 2009** | 70/14 | 42/42 | 48.5 | NR | TACE+HCSI | TACE | ①②③④⑤⑥⑧⑨ |  |
| **Yuan CY 2011** | 28/12 | 20/20 | 52.3±3.5/53.2±3.4 | NR | TACE+HCSI | TACE | ①②④⑤⑥ |  |
| **Jia JY 2016** | 56/39 | 49/46 | 58.4± 8.3/58.1±8.7 | >70 | TACE+HCSI | TACE | ①②④⑥ |  |
| **Wang L 2020** | 55/29 | 42/42 | 59.6±12.2/58.2±13.1 | >60 | TACE+HCSI | TACE | ②④⑤ |  |
| **Liu HQ 2007** | 58/12 | 34/36 | 50.3/51 | 50-90 | TACE+KAI | TACE | ①②③⑤⑥ |  |
| **He W 2009** | 42/40 | 50/32 | 52/49 | NR | TACE+KAI | TACE | ④⑤ |  |
| **Li SR 2013** | 58/22 | 40/40 | 40-71 | NR | TACE+KAI | TACE | ①②③④⑤ |  |
| **Tian H 2013** | 52/12 | 32/32 | 50.3±8.3/53.4±10.5 | >60 | TACE+KAI | TACE | ①②③⑤⑥ |  |
| **Li LH 2016** | 57/23 | 40/40 | 36-73 | NR | TACE+KAI | TACE | ①②③④⑤ |  |
| **Lv DZ 2004** | 61/15 | 38/38 | 49/49 | NR | TACE+KLTI | TACE | ①②③④ |  |
| **Fu TH 2012** | 52/8 | 30/30 | 49.6/49.2 | NR | TACE+XAPI | TACE | ①②③⑤ |  |
| **Meng SH 2021** | 52/28 | 40/40 | 54.76±4.18/53.98±4.43 | NR | TACE+XAPI | TACE | ①②③⑤⑥ |  |
| **Lu YT 2005** | 51/11 | 32/30 | 48.2±0.3/47.8±0.6 | 30-60 | TACE+BOEI | TACE | ①②③④⑤⑥ |  |
| **Wang YH 2014** | 48/8 | 28/28 | 60.5 | NR | TACE+BOEI | TACE | ②③④⑥⑧⑨ |  |
| **Li CT 2017** | 64/36 | 50/50 | 59.8± 6.9/61.4±8.0 | NR | TACE+BOEI | TACE | ①②④⑤⑥ |  |
| Note: M, Male; F, Female; E, Experimental group; C, Control group; NR, not reported;KLTI,Kanglaite injection;ADI:Aidi injection; CKSI, Compound kushen injection;BOEI, Brucea javanica oil emulsion injection;KAI, Kangai injection; HCSI, Huachansu injection; KLTI, Kanglaite injection; SQFZI, Shengqifuzheng injection; XAPI, Xiaoaiping injection.;①CD3^+^T;②CD4^+^T③CD8^+^T;④CD4^+^/CD8^+^T;⑤NK;⑥the clinical effectiveness rate;⑦6-months Survival;⑧1-year Survival; ⑨2-years Survival. | | | | | | | |  |
|  |
